# Crystal structure of 2-(2-bromo­phen­yl)-4-(1*H*-indol-3-yl)-6-(thio­phen-2-yl)pyridine-3-carbo­nitrile

**DOI:** 10.1107/S1600536814017188

**Published:** 2014-08-01

**Authors:** R. Vishnupriya, J. Suresh, Shanmugavel Bharkavi, Subbu Perumal, P. L. Nilantha Lakshman

**Affiliations:** a Department of Physics, The Madura College, Madurai 625 011, India; b Department of Organic Chemistry, School of Chemistry, Madurai Kamaraj University, Madurai 625 021, India; c Department of Food Science and Technology, University of Ruhuna, Mapalana, Kamburupitiya 81100, Sri Lanka

**Keywords:** crystal structure, pyridine-3-carbo­nitrile, hydrogen bonding, π–π stacking

## Abstract

In the title compound, C_24_H_14_BrN_3_S, the dihedral angles between the planes of the pyridine ring and the pendant thio­phene ring, the indole ring system (r.m.s. deviation = 0.022 Å) and the bromo­benzene ring are 9.37 (17), 21.90 (12) and 69.01 (15)°, respectively. The approximate coplanarity of the central ring and the indole ring system is supported by two intra­molecular C—H⋯N inter­actions. In the crystal, inversion dimers linked by pairs of N—H⋯N hydrogen bonds generate *R*_2_^2^(16) loops and the dimers are linked by C—H⋯π and aromatic π–π stacking [shortest centroid–centroid separation = 3.729 (3) Å] into a three-dimensional network.

## Related literature   

For the biological activity of pyridine-3-carbo­nitrile derivatives, see: Kim *et al.* (2005 ▶); Ji *et al.* (2007 ▶); Brandt *et al.* (2010 ▶); El-Sayed *et al.* (2011 ▶).
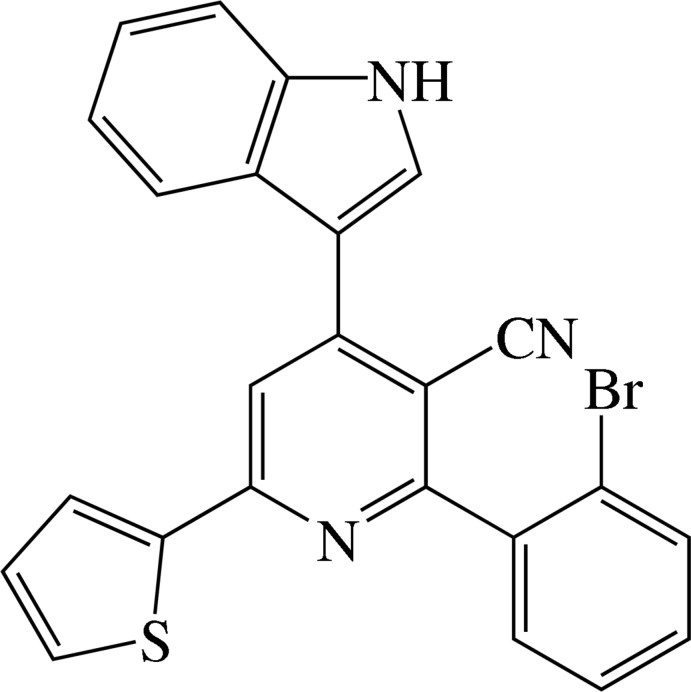


## Experimental   

### Crystal data  

C_24_H_14_BrN_3_S
*M* *_r_* = 456.35
Monoclinic, 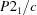

*a* = 10.470 (5) Å
*b* = 21.353 (5) Å
*c* = 9.292 (5) Å
β = 107.710 (5)°
*V* = 1978.9 (15) Å^3^
*Z* = 4
Mo *K*α radiation
μ = 2.20 mm^−1^
*T* = 293 K
0.52 × 0.23 × 0.17 mm

### Data collection  

Bruker Kappa APEXII diffractometer
Absorption correction: multi-scan (*SADABS*; Sheldrick, 1996 ▶) *T*_min_ = 0.958, *T*_max_ = 0.986
17079 measured reflections
4305 independent reflections
2837 reflections with *I* > 2σ(*I*)
*R*_int_ = 0.041

### Refinement  

*R*[*F* ^2^ > 2σ(*F* ^2^)] = 0.046
*wR*(*F* ^2^) = 0.121
*S* = 1.02
4305 reflections
262 parameters
H-atom parameters constrained
Δρ_max_ = 0.48 e Å^−3^
Δρ_min_ = −0.35 e Å^−3^

### 

Data collection: *APEX2* (Bruker, 2004 ▶); cell refinement: *SAINT* (Bruker, 2004 ▶); data reduction: *SAINT*; program(s) used to solve structure: *SHELXS97* (Sheldrick, 2008 ▶); program(s) used to refine structure: *SHELXL97* (Sheldrick, 2008 ▶); molecular graphics: *PLATON* (Spek, 2009 ▶); software used to prepare material for publication: *SHELXL97*.

## Supplementary Material

Crystal structure: contains datablock(s) global, I. DOI: 10.1107/S1600536814017188/hb7260sup1.cif

Structure factors: contains datablock(s) I. DOI: 10.1107/S1600536814017188/hb7260Isup2.hkl

Supporting information file. DOI: 10.1107/S1600536814017188/hb7260Isup3.cml

**Fig. 1.**
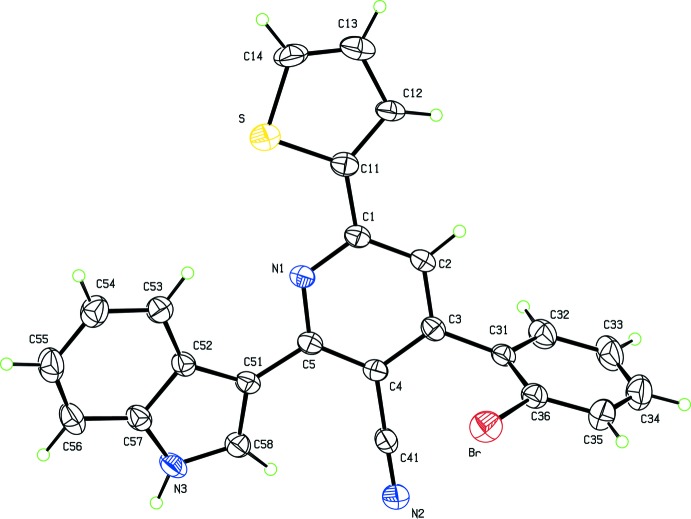
The molecular structure of compound showing 30% probability displacement ellipsoids.

**Fig. 2.**
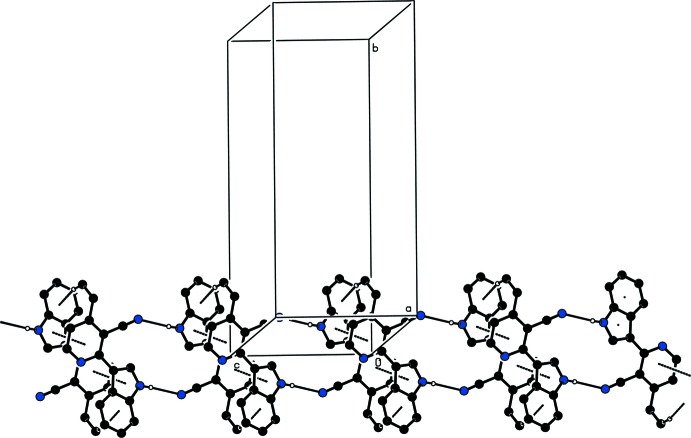
Partial packing view of the compound showing molecules interconnected through a C—H···π stacking interaction (dotted lines; symmetry code: (i) 1/2 - x, 1/2 + y, 1/2 - z)

. DOI: 10.1107/S1600536814017188/hb7260fig1.tif
The mol­ecular structure of compound showing 30% probability displacement ellipsoids.

x y z . DOI: 10.1107/S1600536814017188/hb7260fig2.tif
Partial packing view of the compound showing mol­ecules inter­connected through a C—H⋯π stacking inter­action (dotted lines; symmetry code: (i) 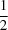 − *x*, 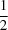 + *y*, 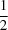 − *z*)

CCDC reference: 1015962

Additional supporting information:  crystallographic information; 3D view; checkCIF report

## Figures and Tables

**Table 1:** Hydrogen-bond geometry (Å, °) *Cg*1 is the centroid of the benzene ring of the indole moiety.

*D*—H⋯*A*	*D*—H	H⋯*A*	*D*⋯*A*	*D*—H⋯*A*
C53—H53⋯N1	0.93	2.58	3.069 (4)	114
C58—H58⋯N2	0.93	2.55	3.278 (4)	135
N3—H3⋯N2^i^	0.86	2.17	3.008 (4)	165
C32—H32⋯*Cg*1^ii^	0.93	2.89	3.761 (4)	157

Symmetry codes: (i) 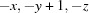; (ii) 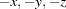.

## supplementary crystallographic information

### S1. Comment

3-Cyanopyridine derivatives have been reported for their wide range of applications such as in antimicrobial, analgesic, anti-hyperglycemic, antiproliferative and antitumor activities (Brandt *et al.*, 2010; El-Sayed *et al.*, 2011; Ji *et al.*, 2007; Kim *et al.*, 2005). As part of our studies in this area, the title compound was investigated.

The deviation of the nitrile atoms (C41,N2) from the mean plane of the pyridine ring system is -0.013 (1) Å and -0.020 (5) Å. The shortening of the C—N distances [1.346 (3) and 1.345 (3) Å] and the opening of the N1–C11–C10 angle [122.83 (2)°] may be attributed to the size of the substituent at C1, correlating well with the values observed in the *ortho*-substituted derivative.

The crystal structure features a intermolecular N—H···N interaction between inverse related molecules generating a graph set ring motif *R*_2_^2^ (16) which are linked into chains through C—H···*Cg*1 interation (*Cg*1 is the centroid of the benzene ring of the indole moiety) and by π···π stacking interaction involving adjacent pyridine and pyrrole rings of the symmetry related molecule at (-*x*, -*y*, -*z*), with a centroid-to-centroid distance of 3.729 (3) Å·(Fig 2).

### S2. Experimental

A mixture of 3-(1*H*-indol-3-yl)-3-oxopropanenitrile 1 (1 mmol), 4,4,4-trifluoro-1-(thiophen-2-yl)butane-1,3-dione 2 (1 mmol) and 2-bromo benzaldehyde 3 (1 mmol) in the presence of ammonium acetate (400 mmol) under solvent-free condition was heated at 110 °C for 7 h. After completion of the reaction (TLC), the reaction mixture was poured into water and extracted with dichloromethane. After removal of the solvent, the residue was chromatographed over silica gel (230–400 mesh) using petroleum ether-ethyl acetate mixture (7:3 *v*/*v*), which afforded the pure compound. Melting point 282°C, yield: 67%.

### S3. Refinement

H atoms were placed at calculated positions and allowed to ride on their carrier atoms with C—H = 0.93–0.98 Å and with *U*_iso_ = 1.2*U*_eq_(C, N) for N, CH_2_ and CH atoms and *U*_iso_ = 1.5*U*_eq_(C) for CH_3_ atoms.

### Crystal data

**Table:**

C_24_H_14_BrN_3_S	*F*(000) = 920
*M**_r_* = 456.35	*D*_x_ = 1.532 Mg m^−^^3^
Monoclinic, *P*2_1_/*c*	Mo *K*α radiation, λ = 0.71073 Å
Hall symbol: -P 2ybc	Cell parameters from 2000 reflections
*a* = 10.470 (5) Å	θ = 2–27°
*b* = 21.353 (5) Å	µ = 2.20 mm^−^^1^
*c* = 9.292 (5) Å	*T* = 293 K
β = 107.710 (5)°	Block, colourless
*V* = 1978.9 (15) Å^3^	0.52 × 0.23 × 0.17 mm
*Z* = 4	

### Data collection

**Table:**

Bruker Kappa APEXII diffractometer	4305 independent reflections
Radiation source: fine-focus sealed tube	2837 reflections with *I* > 2σ(*I*)
Graphite monochromator	*R*_int_ = 0.041
Detector resolution: 0 pixels mm^-1^	θ_max_ = 27.0°, θ_min_ = 1.9°
ω and φ scans	*h* = −12→13
Absorption correction: multi-scan (*SADABS*; Sheldrick, 1996)	*k* = −27→27
*T*_min_ = 0.958, *T*_max_ = 0.986	*l* = −11→11
17079 measured reflections	

### Refinement

**Table:**

Refinement on *F*^2^	Primary atom site location: structure-invariant direct methods
Least-squares matrix: full	Secondary atom site location: difference Fourier map
*R*[*F*^2^ > 2σ(*F*^2^)] = 0.046	Hydrogen site location: inferred from neighbouring sites
*wR*(*F*^2^) = 0.121	H-atom parameters constrained
*S* = 1.02	*w* = 1/[σ^2^(*F*_o_^2^) + (0.0618*P*)^2^ + 0.4334*P*] where *P* = (*F*_o_^2^ + 2*F*_c_^2^)/3
4305 reflections	(Δ/σ)_max_ < 0.001
262 parameters	Δρ_max_ = 0.48 e Å^−^^3^
0 restraints	Δρ_min_ = −0.35 e Å^−^^3^

### Special details

**Table:**

Geometry. All e.s.d.'s (except the e.s.d. in the dihedral angle between two l.s. planes) are estimated using the full covariance matrix. The cell e.s.d.'s are taken into account individually in the estimation of e.s.d.'s in distances, angles and torsion angles; correlations between e.s.d.'s in cell parameters are only used when they are defined by crystal symmetry. An approximate (isotropic) treatment of cell e.s.d.'s is used for estimating e.s.d.'s involving l.s. planes.
Refinement. Refinement of *F*^2^ against ALL reflections. The weighted *R*-factor *wR* and goodness of fit *S* are based on *F*^2^, conventional *R*-factors *R* are based on *F*, with *F* set to zero for negative *F*^2^. The threshold expression of *F*^2^ > σ(*F*^2^) is used only for calculating *R*-factors(gt) *etc*. and is not relevant to the choice of reflections for refinement. *R*-factors based on *F*^2^ are statistically about twice as large as those based on *F*, and *R*- factors based on ALL data will be even larger.

### Fractional atomic coordinates and isotropic or equivalent isotropic displacement parameters (Å^2^)

**Table:**

	*x*	*y*	*z*	*U*_iso_*/*U*_eq_	
C1	0.2640 (3)	0.50193 (12)	0.7417 (3)	0.0386 (7)	
C2	0.3077 (3)	0.44165 (13)	0.7289 (3)	0.0425 (7)	
H2	0.3543	0.4194	0.8149	0.051*	
C3	0.2818 (3)	0.41458 (12)	0.5880 (3)	0.0390 (7)	
C4	0.2078 (3)	0.45002 (12)	0.4630 (3)	0.0368 (6)	
C5	0.1664 (3)	0.51133 (12)	0.4822 (3)	0.0371 (6)	
C11	0.2890 (3)	0.53182 (14)	0.8898 (3)	0.0432 (7)	
C12	0.3376 (3)	0.50426 (15)	1.0315 (3)	0.0481 (8)	
H12	0.3625	0.4624	1.0484	0.058*	
C13	0.3439 (4)	0.54874 (19)	1.1471 (3)	0.0622 (9)	
H13	0.3745	0.5393	1.2496	0.075*	
C14	0.3011 (4)	0.60608 (18)	1.0930 (4)	0.0651 (10)	
H14	0.2995	0.6405	1.1538	0.078*	
C31	0.3330 (3)	0.35097 (13)	0.5742 (3)	0.0402 (7)	
C32	0.2810 (4)	0.30034 (14)	0.6331 (3)	0.0555 (8)	
H32	0.2108	0.3068	0.6733	0.067*	
C33	0.3322 (4)	0.24105 (16)	0.6326 (4)	0.0697 (11)	
H33	0.2958	0.2078	0.6716	0.084*	
C34	0.4351 (5)	0.23060 (17)	0.5759 (4)	0.0736 (11)	
H34	0.4695	0.1904	0.5774	0.088*	
C35	0.4892 (4)	0.27918 (16)	0.5159 (4)	0.0650 (10)	
H35	0.5597	0.2721	0.4766	0.078*	
C36	0.4366 (3)	0.33884 (13)	0.5151 (3)	0.0466 (7)	
C41	0.1792 (3)	0.42163 (13)	0.3172 (3)	0.0451 (7)	
C51	0.0943 (3)	0.55225 (13)	0.3593 (3)	0.0380 (6)	
C52	0.0877 (3)	0.61968 (13)	0.3648 (3)	0.0404 (7)	
C53	0.1419 (3)	0.66595 (14)	0.4726 (3)	0.0497 (8)	
H53	0.1932	0.6550	0.5699	0.060*	
C54	0.1186 (4)	0.72736 (15)	0.4329 (4)	0.0614 (9)	
H54	0.1561	0.7581	0.5041	0.074*	
C55	0.0405 (4)	0.74537 (17)	0.2892 (4)	0.0657 (10)	
H55	0.0253	0.7877	0.2669	0.079*	
C56	−0.0136 (4)	0.70208 (16)	0.1814 (4)	0.0581 (9)	
H56	−0.0650	0.7140	0.0849	0.070*	
C57	0.0104 (3)	0.63913 (14)	0.2198 (3)	0.0451 (7)	
C58	0.0214 (3)	0.53562 (14)	0.2152 (3)	0.0452 (7)	
H58	0.0082	0.4948	0.1791	0.054*	
N1	0.1961 (2)	0.53636 (10)	0.6215 (2)	0.0386 (6)	
N2	0.1568 (3)	0.39821 (12)	0.2015 (3)	0.0630 (8)	
N3	−0.0283 (3)	0.58711 (12)	0.1335 (3)	0.0500 (7)	
H3	−0.0773	0.5870	0.0405	0.060*	
S	0.25060 (12)	0.60852 (4)	0.90204 (10)	0.0706 (3)	
Br	0.51614 (4)	0.404354 (16)	0.43368 (4)	0.06285 (16)	

### Atomic displacement parameters (Å^2^)

**Table:**

	*U*^11^	*U*^22^	*U*^33^	*U*^12^	*U*^13^	*U*^23^
C1	0.0378 (18)	0.0460 (16)	0.0330 (14)	−0.0027 (13)	0.0123 (13)	0.0018 (13)
C2	0.049 (2)	0.0473 (17)	0.0288 (14)	0.0025 (14)	0.0084 (13)	0.0054 (12)
C3	0.0397 (18)	0.0415 (15)	0.0365 (15)	−0.0016 (13)	0.0128 (13)	0.0067 (12)
C4	0.0403 (18)	0.0393 (14)	0.0298 (14)	−0.0037 (13)	0.0094 (12)	0.0000 (12)
C5	0.0362 (17)	0.0420 (15)	0.0330 (14)	−0.0041 (13)	0.0103 (12)	0.0019 (12)
C11	0.0382 (18)	0.0517 (17)	0.0380 (16)	−0.0058 (14)	0.0093 (13)	−0.0024 (13)
C12	0.054 (2)	0.0600 (18)	0.0271 (14)	−0.0014 (15)	0.0083 (14)	−0.0045 (14)
C13	0.059 (2)	0.088 (3)	0.0347 (17)	−0.005 (2)	0.0078 (16)	−0.0043 (17)
C14	0.076 (3)	0.075 (2)	0.0419 (18)	−0.001 (2)	0.0139 (18)	−0.0232 (17)
C31	0.0467 (19)	0.0402 (15)	0.0285 (14)	0.0000 (13)	0.0038 (13)	0.0009 (12)
C32	0.062 (2)	0.0500 (19)	0.0537 (19)	−0.0032 (16)	0.0163 (17)	0.0094 (15)
C33	0.087 (3)	0.0421 (19)	0.071 (2)	−0.0048 (19)	0.012 (2)	0.0172 (17)
C34	0.086 (3)	0.046 (2)	0.080 (3)	0.011 (2)	0.013 (2)	0.0019 (18)
C35	0.070 (3)	0.057 (2)	0.066 (2)	0.0158 (18)	0.0176 (19)	−0.0031 (18)
C36	0.051 (2)	0.0464 (17)	0.0392 (16)	−0.0005 (15)	0.0082 (15)	0.0002 (13)
C41	0.054 (2)	0.0375 (15)	0.0383 (17)	0.0044 (14)	0.0051 (14)	0.0058 (14)
C51	0.0384 (18)	0.0446 (15)	0.0318 (14)	−0.0001 (13)	0.0119 (13)	0.0035 (12)
C52	0.0407 (18)	0.0457 (15)	0.0389 (16)	0.0046 (14)	0.0182 (14)	0.0090 (13)
C53	0.056 (2)	0.0463 (17)	0.0477 (18)	0.0001 (15)	0.0179 (16)	0.0005 (14)
C54	0.072 (3)	0.0451 (18)	0.073 (2)	−0.0003 (17)	0.031 (2)	0.0008 (17)
C55	0.076 (3)	0.0490 (19)	0.078 (3)	0.0099 (18)	0.032 (2)	0.0204 (19)
C56	0.057 (2)	0.061 (2)	0.058 (2)	0.0113 (18)	0.0199 (17)	0.0262 (18)
C57	0.0436 (19)	0.0540 (18)	0.0409 (16)	0.0052 (15)	0.0177 (14)	0.0119 (14)
C58	0.045 (2)	0.0494 (17)	0.0402 (16)	0.0037 (14)	0.0111 (14)	0.0024 (14)
N1	0.0424 (15)	0.0416 (13)	0.0316 (12)	−0.0021 (11)	0.0107 (11)	0.0006 (10)
N2	0.089 (2)	0.0508 (16)	0.0405 (15)	0.0127 (14)	0.0065 (15)	−0.0049 (12)
N3	0.0488 (18)	0.0617 (17)	0.0330 (13)	0.0058 (13)	0.0024 (12)	0.0085 (12)
S	0.0982 (9)	0.0602 (5)	0.0497 (5)	0.0089 (5)	0.0167 (5)	−0.0073 (4)
Br	0.0616 (3)	0.0661 (3)	0.0687 (3)	−0.00767 (17)	0.0314 (2)	0.00167 (16)

### Geometric parameters (Å, º)

**Table:**

C1—N1	1.345 (3)	C33—H33	0.9300
C1—C2	1.383 (4)	C34—C35	1.378 (5)
C1—C11	1.466 (4)	C34—H34	0.9300
C2—C3	1.380 (4)	C35—C36	1.387 (4)
C2—H2	0.9300	C35—H35	0.9300
C3—C4	1.405 (4)	C36—Br	1.899 (3)
C3—C31	1.480 (4)	C41—N2	1.143 (4)
C4—C5	1.408 (4)	C51—C58	1.371 (4)
C4—C41	1.431 (4)	C51—C52	1.443 (4)
C5—N1	1.346 (3)	C52—C53	1.399 (4)
C5—C51	1.454 (4)	C52—C57	1.407 (4)
C11—C12	1.390 (4)	C53—C54	1.364 (4)
C11—S	1.698 (3)	C53—H53	0.9300
C12—C13	1.420 (4)	C54—C55	1.391 (5)
C12—H12	0.9300	C54—H54	0.9300
C13—C14	1.347 (5)	C55—C56	1.354 (5)
C13—H13	0.9300	C55—H55	0.9300
C14—S	1.691 (4)	C56—C57	1.394 (4)
C14—H14	0.9300	C56—H56	0.9300
C31—C36	1.381 (4)	C57—N3	1.358 (4)
C31—C32	1.395 (4)	C58—N3	1.347 (4)
C32—C33	1.376 (5)	C58—H58	0.9300
C32—H32	0.9300	N3—H3	0.8600
C33—C34	1.354 (6)		
			
N1—C1—C2	122.8 (2)	C35—C34—H34	119.8
N1—C1—C11	116.1 (2)	C34—C35—C36	118.8 (4)
C2—C1—C11	121.1 (2)	C34—C35—H35	120.6
C3—C2—C1	119.8 (3)	C36—C35—H35	120.6
C3—C2—H2	120.1	C31—C36—C35	121.9 (3)
C1—C2—H2	120.1	C31—C36—Br	120.9 (2)
C2—C3—C4	117.3 (3)	C35—C36—Br	117.2 (3)
C2—C3—C31	119.7 (2)	N2—C41—C4	179.1 (3)
C4—C3—C31	123.1 (2)	C58—C51—C52	105.9 (2)
C3—C4—C5	120.6 (2)	C58—C51—C5	127.8 (3)
C3—C4—C41	117.3 (2)	C52—C51—C5	126.2 (2)
C5—C4—C41	122.0 (2)	C53—C52—C57	117.9 (3)
N1—C5—C4	120.1 (2)	C53—C52—C51	135.8 (3)
N1—C5—C51	115.4 (2)	C57—C52—C51	106.3 (2)
C4—C5—C51	124.5 (2)	C54—C53—C52	119.0 (3)
C12—C11—C1	127.9 (3)	C54—C53—H53	120.5
C12—C11—S	111.8 (2)	C52—C53—H53	120.5
C1—C11—S	120.3 (2)	C53—C54—C55	122.0 (3)
C11—C12—C13	110.6 (3)	C53—C54—H54	119.0
C11—C12—H12	124.7	C55—C54—H54	119.0
C13—C12—H12	124.7	C56—C55—C54	120.9 (3)
C14—C13—C12	113.1 (3)	C56—C55—H55	119.6
C14—C13—H13	123.5	C54—C55—H55	119.6
C12—C13—H13	123.5	C55—C56—C57	117.8 (3)
C13—C14—S	112.5 (3)	C55—C56—H56	121.1
C13—C14—H14	123.8	C57—C56—H56	121.1
S—C14—H14	123.8	N3—C57—C56	129.7 (3)
C36—C31—C32	117.3 (3)	N3—C57—C52	107.8 (3)
C36—C31—C3	123.8 (3)	C56—C57—C52	122.4 (3)
C32—C31—C3	118.8 (3)	N3—C58—C51	110.1 (3)
C33—C32—C31	120.8 (3)	N3—C58—H58	124.9
C33—C32—H32	119.6	C51—C58—H58	124.9
C31—C32—H32	119.6	C1—N1—C5	119.4 (2)
C34—C33—C32	120.7 (3)	C58—N3—C57	109.9 (3)
C34—C33—H33	119.7	C58—N3—H3	125.1
C32—C33—H33	119.7	C57—N3—H3	125.1
C33—C34—C35	120.5 (3)	C14—S—C11	92.04 (16)
C33—C34—H34	119.8		
			
N1—C1—C2—C3	0.0 (5)	C34—C35—C36—C31	0.8 (5)
C11—C1—C2—C3	179.6 (3)	C34—C35—C36—Br	179.2 (3)
C1—C2—C3—C4	−1.7 (4)	N1—C5—C51—C58	−161.3 (3)
C1—C2—C3—C31	177.8 (3)	C4—C5—C51—C58	20.3 (5)
C2—C3—C4—C5	2.1 (4)	N1—C5—C51—C52	20.1 (4)
C31—C3—C4—C5	−177.3 (3)	C4—C5—C51—C52	−158.3 (3)
C2—C3—C4—C41	−179.0 (3)	C58—C51—C52—C53	−177.4 (3)
C31—C3—C4—C41	1.6 (4)	C5—C51—C52—C53	1.5 (5)
C3—C4—C5—N1	−0.8 (4)	C58—C51—C52—C57	−0.4 (3)
C41—C4—C5—N1	−179.7 (3)	C5—C51—C52—C57	178.4 (3)
C3—C4—C5—C51	177.5 (3)	C57—C52—C53—C54	−0.4 (4)
C41—C4—C5—C51	−1.4 (4)	C51—C52—C53—C54	176.3 (3)
N1—C1—C11—C12	169.4 (3)	C52—C53—C54—C55	1.2 (5)
C2—C1—C11—C12	−10.2 (5)	C53—C54—C55—C56	−1.3 (5)
N1—C1—C11—S	−7.5 (4)	C54—C55—C56—C57	0.7 (5)
C2—C1—C11—S	172.9 (2)	C55—C56—C57—N3	−178.0 (3)
C1—C11—C12—C13	−178.3 (3)	C55—C56—C57—C52	0.0 (5)
S—C11—C12—C13	−1.2 (4)	C53—C52—C57—N3	178.3 (3)
C11—C12—C13—C14	0.5 (4)	C51—C52—C57—N3	0.7 (3)
C12—C13—C14—S	0.5 (4)	C53—C52—C57—C56	−0.2 (4)
C2—C3—C31—C36	−108.6 (3)	C51—C52—C57—C56	−177.8 (3)
C4—C3—C31—C36	70.8 (4)	C52—C51—C58—N3	0.1 (3)
C2—C3—C31—C32	66.9 (4)	C5—C51—C58—N3	−178.8 (3)
C4—C3—C31—C32	−113.7 (3)	C2—C1—N1—C5	1.3 (4)
C36—C31—C32—C33	0.4 (5)	C11—C1—N1—C5	−178.3 (2)
C3—C31—C32—C33	−175.3 (3)	C4—C5—N1—C1	−0.9 (4)
C31—C32—C33—C34	0.5 (5)	C51—C5—N1—C1	−179.3 (2)
C32—C33—C34—C35	−0.8 (6)	C51—C58—N3—C57	0.4 (4)
C33—C34—C35—C36	0.2 (6)	C56—C57—N3—C58	177.6 (3)
C32—C31—C36—C35	−1.1 (4)	C52—C57—N3—C58	−0.6 (4)
C3—C31—C36—C35	174.4 (3)	C13—C14—S—C11	−1.0 (3)
C32—C31—C36—Br	−179.4 (2)	C12—C11—S—C14	1.3 (3)
C3—C31—C36—Br	−3.9 (4)	C1—C11—S—C14	178.6 (3)

### Hydrogen-bond geometry (Å, º)

Cg1 is the centroid of the benzene ring of the indole moiety.

**Table:**

*D*—H···*A*	*D*—H	H···*A*	*D*···*A*	*D*—H···*A*
C53—H53···N1	0.93	2.58	3.069 (4)	114
C58—H58···N2	0.93	2.55	3.278 (4)	135
N3—H3···N2^i^	0.86	2.17	3.008 (4)	165
C32—H32···*Cg*1^ii^	0.93	2.89	3.761 (4)	157

Symmetry codes: (i) −*x*, −*y*+1, −*z*; (ii) −*x*, −*y*, −*z*.
