# Supplementary figures and images for: Crystal structure of 2-(2-bromo­phen­yl)-4-(1H-indol-3-yl)-6-(thio­phen-2-yl)pyridine-3-carbo­nitrile
Source: Acta Crystallogr Sect E Struct Rep Online. 2014 Aug 1;70(Pt 9):o968–9. doi: 10.1107/S1600536814017188 (PMC4186133; doi:10.1107/S1600536814017188)

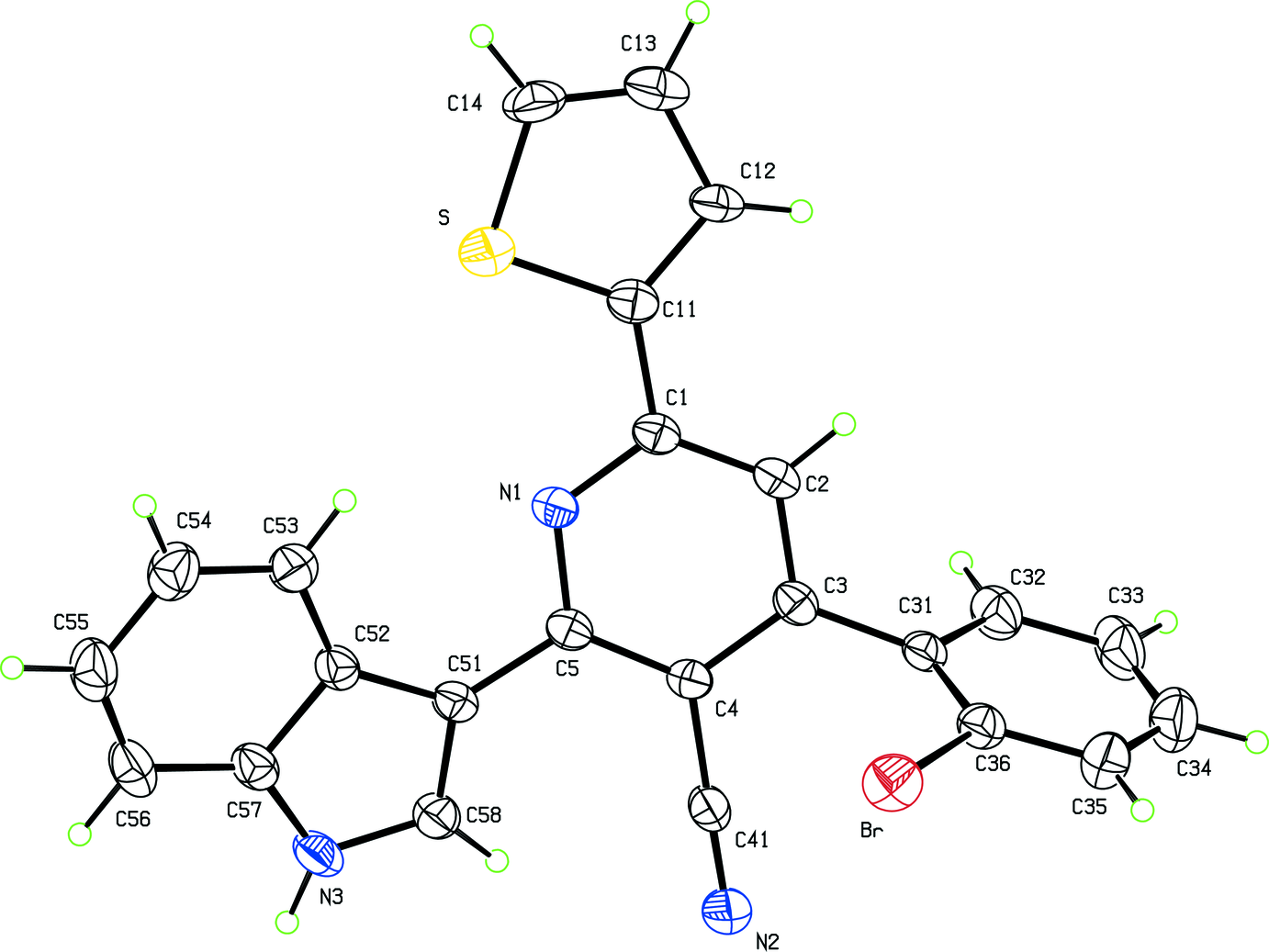

Supplement: Supplementary file 4 [file e-70-0o968-fig1.tif]

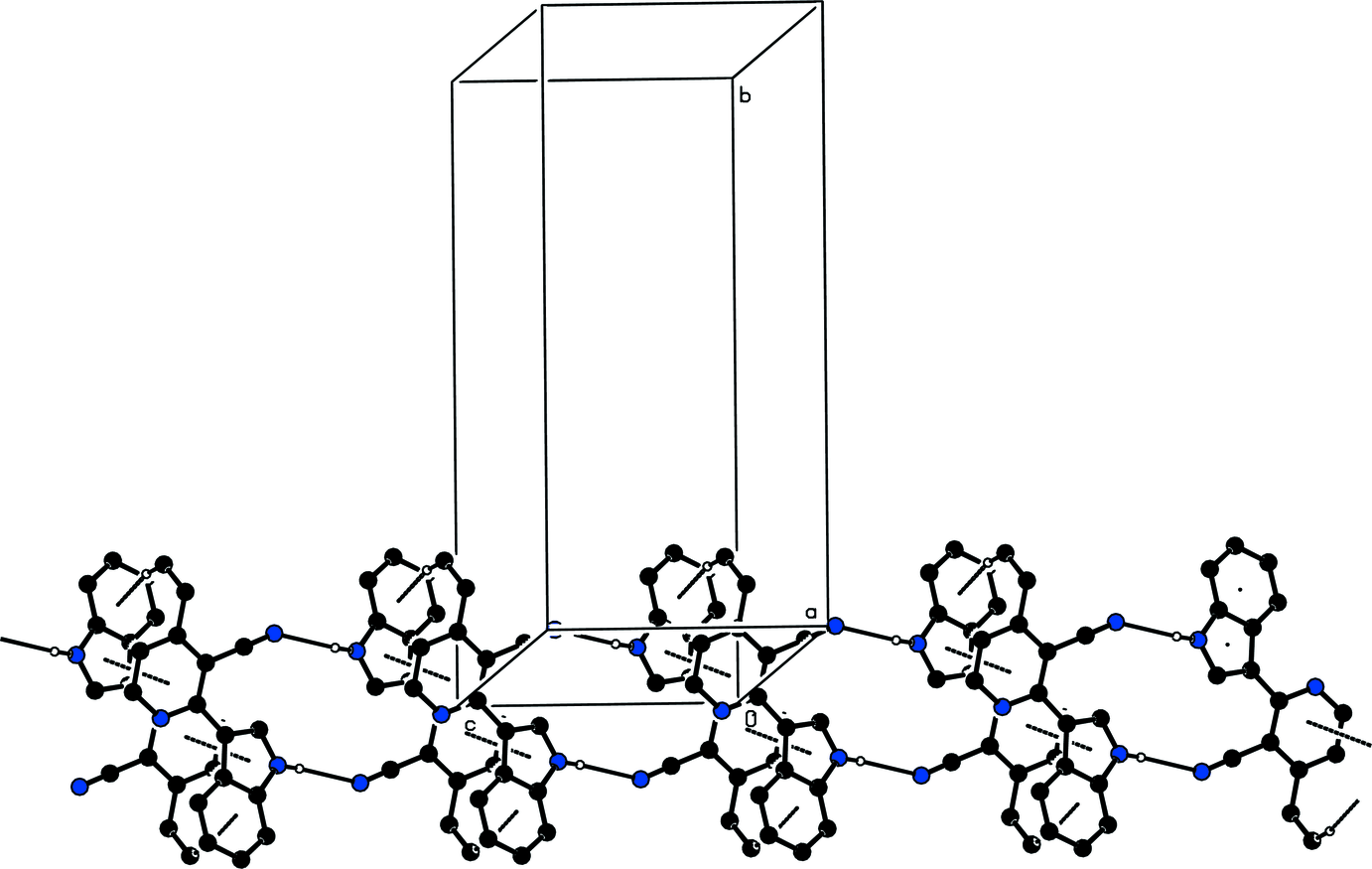

Supplement: Supplementary file 5 [file e-70-0o968-fig2.tif]
